# MPRAGE_*like*_: A novel approach to generate T1w images from multi‐contrast gradient echo images for brain segmentation

**DOI:** 10.1002/mrm.30453

**Published:** 2025-02-04

**Authors:** Marc‐Antoine Fortin, Rüdiger Stirnberg, Yannik Völzke, Laurent Lamalle, Eberhard Pracht, Daniel Löwen, Tony Stöcker, Pål Erik Goa

**Affiliations:** ^1^ Department of Physics Norwegian University of Science and Technology Trondheim Trøndelag Norway; ^2^ German Center for Neurodegenerative Diseases (DZNE) Bonn Germany; ^3^ GIGA‐Cyclotron Research Centre‐In Vivo Imaging University of Liège Liège Belgium; ^4^ Department of Physics and Astronomy University of Bonn Bonn Germany; ^5^ Department of Radiology and Nuclear Medicine St. Olavs Hospital HF Trondheim Norway

**Keywords:** brain segmentation, multi‐contrast, multi‐parameter mapping, neuroimaging, synthetic MPRAGE, UHF‐MRI

## Abstract

**Purpose:**

Brain segmentation and multi‐parameter mapping (MPM) are important steps in neurodegenerative disease characterization. However, acquiring both a high‐resolution T1w sequence like MPRAGE (standard input to brain segmentation) and an MPM in the same neuroimaging protocol increases scan time and patient discomfort, making it difficult to combine both in clinical examinations.

**Methods:**

A novel approach to synthesize T1w images from MPM images, named MPRAGE_*like*_, is proposed and compared to the standard technique used to produce synthetic MPRAGE images (*syn*MPRAGE). Twenty‐three healthy subjects were scanned with the same imaging protocol at three different 7T sites using universal parallel transmit RF pulses. SNR, CNR, and automatic brain segmentation results from both MPRAGE_*like*_ and *syn*MPRAGE were compared against an acquired MPRAGE.

**Results:**

The proposed MPRAGE_*like*_ technique produced higher SNR values than *syn*MPRAGE for all regions evaluated while also having higher CNR values for subcortical structures. MPRAGE was still the image with the highest SNR values overall. For automatic brain segmentation, MPRAGE_*like*_ outperformed *syn*MPRAGE when compared to MPRAGE (median Dice Similarity Coefficient of 0.90 versus 0.29 and Average Asymmetric Surface Distance of 0.33 versus 2.93 mm, respectively), in addition to being simple, flexible, and considerably more robust to low image quality than *syn*MPRAGE.

**Conclusion:**

The MPRAGE_*like*_ technique can provide a better and more reliable alternative to *syn*MPRAGE as a substitute for MPRAGE, especially when automatic brain segmentation is of interest and scan time is limited.

## 1 INTRODUCTION

Magnetization‐Prepared Rapid Acquisition of Gradient Echoes (MPRAGE) has excellent white and gray matter (WM‐GM) contrast and short acquisition time.^1^ It is therefore the preferred anatomical reference image in most neuroimaging studies. *FreeSurfer*,^2^ which provides automated brain segmentation from T1w MPRAGE images at 1.5T and 3T, has been widely used in large‐scale neuroimaging projects^3^ and, recently, a Deep Learning (DL)‐based version called *FastSurferVINN* ^4^ has been proposed, which drastically reduces computational time.

In the past decade, Ultra‐High Field Magnetic Resonance Imaging (UHF‐MRI) (i.e., ≥ 7T) has become more widely used in neuroimaging studies due to its higher SNR, contrast, and spatial resolution.^5^ However, UHF‐MRI suffers from larger transmit RF inhomogeneities than those observed at lower field strengths due to the shorter RF wavelength at 7T.^6^ This is especially problematic for the RF field used for excitation ($B_{1}^{+}$), resulting in considerable inhomogeneity in both signal intensity and tissue contrast across the field of view (FOV).^7^ To compensate for this effect, a more time‐consuming MP2RAGE sequence is often utilized at UHF.^8^ The MP2RAGE sequence acquires two images, one at a short and one at a long inversion time, which are combined to calculate a third and more homogeneous image often referred to as the UNI image. A regularization term λ can be added to the calculation of the UNI image to suppress background noise at the cost of re‐introducing a small amount of inhomogeneity.^9^ Additionally, a simpler alternative to MP2RAGE proposes to use a non‐interleaved GRE acquisition to remove the PD, $T_{2}^{*}$, and receive sensitivity weightings on MPRAGE images at 7T.^10^

Recent progress in RF pulse design has enabled parallel transmission (pTx) RF pulses that considerably reduce the amount of signal inhomogeneities at UHF. One of the most promising pTx techniques applies calibration‐free, universal pTx‐pulses (UP),^11^ which are pre‐computed pTx‐pulses optimized offline across multiple subjects, removing the need for time‐consuming pTx‐pulse optimization during each MR exam.

Advanced neuroimaging protocols such as applied in Alzheimer's^12^ and Parkinson's Diseases^13^ research has recently been extended to include quantitative parameter mapping methods, such as Multi‐Parameter Mapping (MPM)^14^ and Variable Flip Angle (VFA) imaging.^15^ These methods acquire multi‐contrast spoiled Gradient Echo (GRE) images in relatively short time for the calculation of quantitative maps (qMaps)^16^ and have become valuable tools for multi‐center neuroimaging studies.^17^, ^18^, ^19^ However, MPRAGE is usually still included due to its high importance as an anatomical reference and basis for automatic segmentation.

When combining anatomical multi‐contrast imaging and quantitative parameter mapping, neuroimaging protocols can become prohibitively long. To avoid the MPRAGE acquisition, several techniques to create “synthetic” T1w images have been proposed.^20^, ^21^, ^22^, ^23^ One of them proposes to derive synthetic MPRAGE (*syn*MPRAGE) images from a combination of R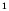, Proton Density (PD), and R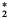 maps.^21^ A recent study using this technique has shown the clinical relevance of *syn*MPRAGE images in spinal cord neurodegeneration characterization at 3T.^24^ Furthermore, techniques using DL have been proposed to synthesize MPRAGE images from multi‐echo (ME) GRE images^22^ or even from any MR image.^23^ In Reference 22, high correlation of segmentation and susceptibility results produced from using either the synthetic T1w or MPRAGE images was presented.

However, all synthetic T1w techniques proposed in the literature require either (1) the computation of qMaps beforehand or (2) DL training, both of which are time‐consuming and require highly specialized processing pipelines. Additionally, the usage of synthetic T1w contrasts at UHF‐MRI has been quite sparse and has shown unsatisfactory results when tested,^25^ necessitating novel approaches to be developed at UHF‐MRI. To the best of our knowledge, no technique has been proposed thus far to synthesize T1w contrast images directly from MPM images without the need for advanced preprocessing.

In this work, we propose MPRAGE_*like*_, a novel and straightforward method to synthesize T1w images from MPM images acquired with 3D‐EPI readouts. We test it against the most commonly used technique to generate *syn*MPRAGE images, and we compare the results of automatic brain segmentation based on MPRAGE_*like*_ images with those obtained from UP‐MPRAGE acquisitions. All results reported were obtained at 7T. The name MPRAGE_*like*_ is inspired by its visual resemblance to the acquired MPRAGE and its primary intended use for automatic segmentation in neuroimaging pipelines, despite them being produced in entirely different ways.

## 2 METHODS

### 2.1 Subjects

A total of 23 healthy volunteers were recruited (male/female: 16/7, age: 30.2 ± 7.5 years old) at three partner sites; nine at site A (NTNU, Trondheim, Norway), ten at site B (DZNE, Bonn, Germany), and four at site C (GIGA‐CRC, Liège, Belgium). The study was approved by the local review boards of each site, and volunteers signed a written informed consent form before scanning.

### 2.2 Image acquisition

Images were acquired on Siemens MAGNETOM Terra (sites A and C) and MAGNETOM 7TPlus (site B) scanners. All sites used identical 8Tx/32Rx head coils (Nova Medical, Wilmington, MA) in pTx mode. The imaging protocol included three contrast acquisitions for MPM, MPRAGE and a flip‐angle map. All scans used UPs created from a database of $B_{0}$ and $B_{1}^{+}$ maps acquired from 10/10/9 subjects at sites A/B/C in a preceding study.

The MPM protocol used a multi‐echo (ME) skipped‐CAIPI 3D‐EPI sequence^16^, ^26^ with universal GRAPE^27^ binomial‐121 water excitation (1.54 ms pulse duration) to acquire images with MTw, PDw, and T1w contrasts. The scan parameters were the following: voxel size = (0.6 mm)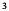, TE = 4.7, 11.8, 18.9, 26.0 ms for four TEs, 80% phase partial Fourier sampling, $1\times 3_{z1}$ CAIPIRINHA parallel imaging,^28^ EPI factor of 4, MTw/PDw/T1w nominal flip angle = 6°/5°/25°, TR = 50.5/34.0/34.0 ms, TA = 6:00/4:05/4:05 min (total scan time 14:10 min). The MTw scan used PUSHUP pTx saturation pulses,^29^ which is a UP variation of previously proposed PUSH saturation^30^ composed of two Gaussian‐shaped sub‐pulses with a duration of 5 ms each with a target $B_{1,\mathrm{rms}}=1.7$ μT.

The scan parameters for the MPRAGE were the following: voxel size = (0.6 mm)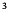, TE = 2.14 ms, $1\times 2_{z1}$ CAIPIRINHA parallel imaging, elliptical sampling, nominal flip angle = 5°, TI = 1100 ms, TR = 2700 ms, TA = 7:24 min.

The Actual Flip Angle Imaging (AFI)^31^ technique was used to map the local flip angle of the MPM excitation pulse. Scan parameters were the following: voxel size = (4.0 mm)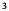, TE = 2.04 ms, $2\times 2_{z1}$ CAIPIRINHA parallel imaging, nominal flip angle = 45°, TR = 20/200 ms, TA = 1:50 min.

### 2.3 Data processing

The MPM magnitude images and $B_{1}^{+}$ map were processed using the hMRI toolbox (v0.6.1)^32^ embedded in SPM12^33^ to first coregister the MPM images with a rigid registration step, then calculate the $B_{1}^{+}$‐corrected R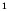, PD, MT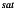, and R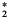 maps. Additionally, the MPRAGE images were rigidly coregistered to the 1st TE MPM magnitude images to correct for inter‐scan motion from subjects. More details about the individual steps are available in Reference 32.

### 2.4 MPRAGE_*like*_

The raw T1w MPM images do not provide sufficient morphometric details to be used for visual assessment or automatic brain segmentation. On the other hand, PDw and MTw contrasts in the MPM protocol provide an approximately “*inverted*” contrast compared to the T1w. The rationale behind calculating MPRAGE_*like*_ images is (1) to increase the T1w contrast‐to‐noise ratio (CNR) by dividing the T1w image by the approximately “*inverted*” contrast images acquired in the MPM protocol, (2) thereby reducing receive bias and residual transmit bias, and (3) canceling out $T_{2}^{*}$ bias in accordance with Reference 8, without the need to calculate qMaps. The following three equations for generating MPRAGE_*like*_ images were tested:

(1) $$MPRAGE_{like,all}\;=\;\frac{T_{1w}-\lambda }{0.5\cdot (PD_{w}+MT_{w})+\lambda },$$

(2) $$MPRAGE_{like,MT}\;=\;\frac{T_{1w}-\lambda }{MT_{w}+\lambda },$$

(3) $$MPRAGE_{like,PD}\;=\;\frac{T_{1w}-\lambda }{PD_{w}+\lambda },$$

where PDw, MTw, and T1w are the 1st TE magnitude MPM images and λ the regularization term. All intensity values < 0 and > 500 were set to 0.

Following the standard MPM theory and derivations of signal equations as described in References 14 and 34 (and ignoring λ), we can obtain the following receive bias‐free approximations for the three signal equations:

(4) $$\begin{array}{ll} & MPRAGE_{like,all}\\ & ≅\frac{\frac{\alpha_{\mathrm{no}m_{T1}}TR_{T1}}{\alpha_{\mathrm{no}m_{T1}}^{2}+2\cdot TR_{T1}\cdot R_{\text{1app}}}}{0.5\times \left(\frac{\alpha_{\mathrm{no}m_{\mathrm{MT}}}TR_{\mathrm{MT}}}{\alpha_{\mathrm{no}m_{\mathrm{MT}}}^{2}+2\cdot (\delta_{\text{app}}+TR_{\mathrm{MT}}\cdot R_{\text{1app}})}+\frac{\alpha_{\mathrm{no}m_{\mathrm{PD}}}TR_{\mathrm{PD}}}{\alpha_{\mathrm{no}m_{\mathrm{PD}}}^{2}+2\cdot TR_{\mathrm{PD}}\cdot R_{\text{1app}}}\right)}, \end{array}$$

(5) $$\begin{array}{ll} & MPRAGE_{like,MT}\\ & ≅\left(\frac{\alpha_{\mathrm{no}m_{T1}}TR_{T1}}{\alpha_{\mathrm{no}m_{\mathrm{MT}}}TR_{\mathrm{MT}}}\right)\cdot \left(\frac{\alpha_{\mathrm{no}m_{\mathrm{MT}}}^{2}+2\cdot (\delta_{\text{app}}+TR_{\mathrm{MT}}\cdot R_{\text{1app}})}{\alpha_{\mathrm{no}m_{T1}}^{2}+2\cdot TR_{T1}\cdot R_{\text{1app}}}\right) \end{array}$$

(6) $$\begin{array}{l} MPRAGE_{like,PD}≅\left(\frac{\alpha_{\mathrm{no}m_{T1}}}{\alpha_{\mathrm{no}m_{\mathrm{PD}}}}\right)\cdot \left(\frac{\alpha_{\mathrm{no}m_{\mathrm{PD}}}^{2}+2\cdot TR_{\mathrm{PD}}\cdot R_{\text{1app}}}{\alpha_{\mathrm{no}m_{T1}}^{2}+2\cdot TR_{T1}\cdot R_{\text{1app}}}\right), \end{array}$$

where $\alpha_{\mathrm{no}m_{X}}$ and $TR_{X}$ are the nominal flip angle and TR values of the T1w, MTw, and PDw contrasts, respectively, $R_{\text{1app}}$, the apparent longitudinal relaxation rate, and $\delta_{\text{app}}$, the apparent MT saturation. For more details about the derivation of Equations (4), (5), and (6), we refer the reader to Supporting Information. Note that Equations (4) through (6) are free of receive bias, but they are not free of potential signal inhomogeneities, as both $R_{\text{1app}}$ and $\delta_{\text{app}}$ depend on $B_{1}^{+}$.

### 2.5 *syn*MPRAGE

For the calculation of *syn*MPRAGE images, the $B_{1}^{+}$‐corrected $R_{1}$ map computed by the hMRI toolbox in addition to the sequence parameters of the acquired MPRAGE protocol (provided in section 2.2) were used. More precisely, these values were substituted in eq. 3a of Reference 21 in addition to the PDw with both receive and transmit bias contributions being removed by assuming a constant value of 1 ($M_{0}$ = 1, RP = 1, and $B_{1}$ = 1 in eq. 3a of Reference 21). This version of the *syn*MPRAGE equation was selected to produce a superior optical contrast^21^ and to create a “pure T1w”, free of PD, $T_{2}^{*}$, and inhomogeneities like for the MPRAGE_*like*_.

**FIGURE 1:**
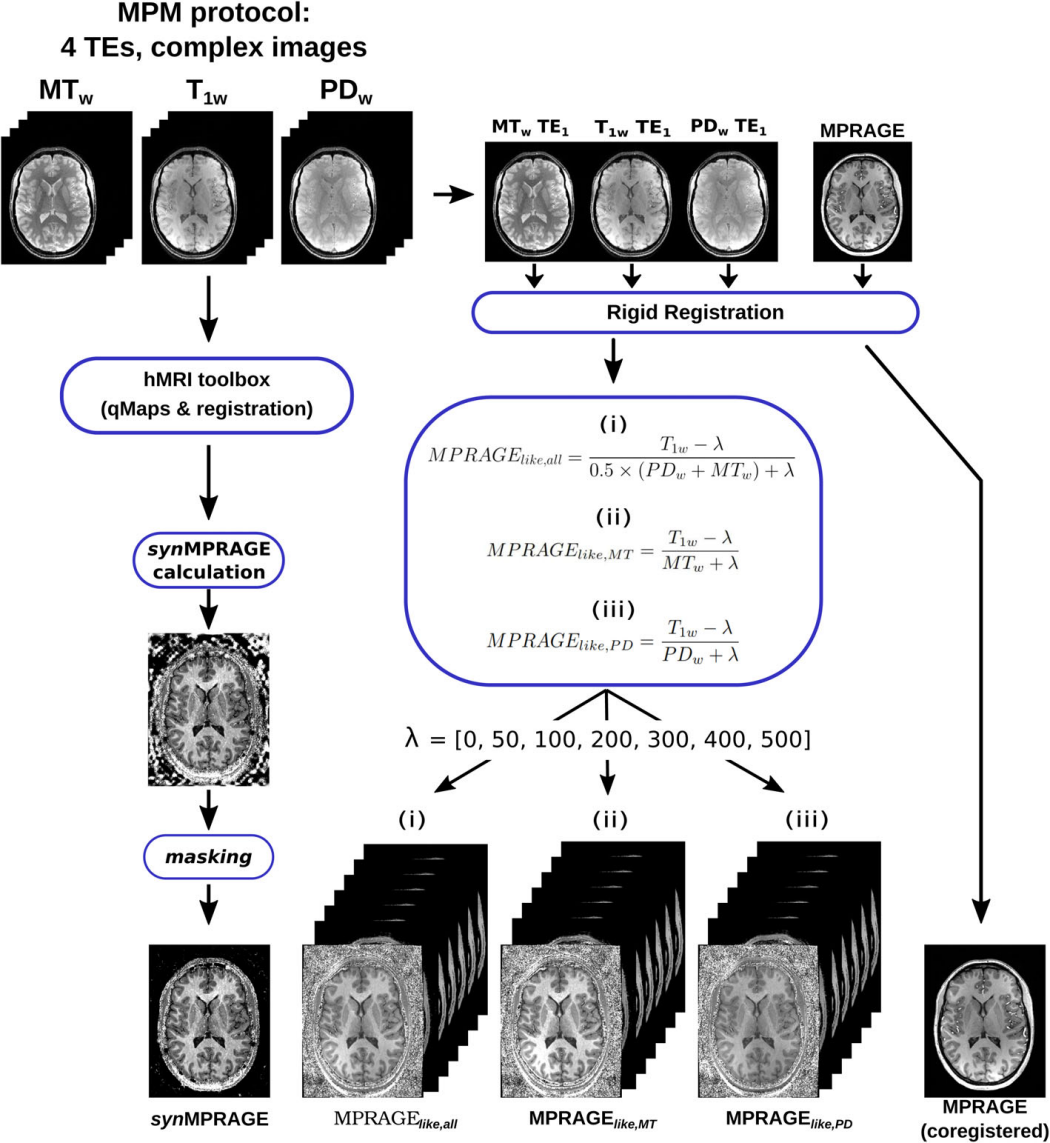
Processing pipeline used in this study to compute *syn*MPRAGE and MPRAGE_*like*_ images. The 1st TE MPM images from each contrast were first registered and then combined to produce the MPRAGE_*like,all*_ (i), MPRAGE*like, MT* (ii), and MPRAGE_*like,PD*_ (iii) images with different λ values. In order to compute the $R_{1}$ map necessary for the creation of *syn*MPRAGE images, all 12 magnitude volumes were given as inputs to the hMRI toolbox (v0.6.1).^32^ The MPRAGE images were also coregistered through hMRI to correct for inter‐scan motion. The background noise in the *syn*MPRAGE was partially masked by removing negative outliers from the PD map, thresholding it with Otsu's method^35^ and applying morphological operations (dilation and closing) to create a mask applied on the $R_{1}$ map. More details about the *syn*MPRAGE calculation are given in Reference 21.

The complete processing pipeline used in this study is shown in Figure 1.

### 2.6 Image analysis

First, to assess the impact of the value of the regularization term λ on the MPRAGE_*like*_ images, the following λ values were tested: 0, 50, 100, 200, 300, 400, and 500. For all λ values and MPRAGE_*like*_ equations, the voxel‐wise relative difference in signal intensity (Δs/s) between the λ = 0 image and other λ values was computed with the following equation:

(7) $$\Delta s/s\;=\;100\times \frac{MPRAGE_{like}(\lambda )-MPRAGE_{like}(\lambda =0)}{MPRAGE_{like}(\lambda =0)},$$

where MPRAGE_*like*_ (λ) is one of the regularized images and MPRAGE_*like*_ (λ = 0) is the non‐regularized image.

Moreover, to determine an optimal λ, the Structural Similarity Index Metric (SSIM)^36^ was computed for each MPRAGE_*like*_ equation and λ with the MPRAGE as the reference image for all subjects in this study. The SSIM was calculated with the following equation:

(8) $$\begin{array}{cc} \text{SSIM}(x,y) & =\frac{\left(2\mu_{x}\mu_{y}+C_{1})(2\sigma_{x}\sigma_{y}+C_{2}\right)}{\left(\mu_{x}^{2}+\mu_{y}^{2}+C_{1})(\sigma_{x}^{2}+\sigma_{y}^{2}+C_{2}\right)}\\ & \;\times \frac{\left(cov_{xy}+C_{3}\right)}{\left(\sigma_{x}\sigma_{y}+C_{3}\right)}, \end{array}$$

where x and y are the two compared images, $\mu_{x}$ and $\mu_{y}$ are the mean values of the images x and y, $\sigma_{x}$ and $\sigma_{y}$ the standard deviation of images x and y, $cov_{xy}$ the covariance between images x and y, and $C_{1}$, $C_{2}$, and $C_{3}$ are constants for stabilization in case of weak denominators automatically set for each image as explained in Reference 36.

Once the value of λ that yielded the largest agreement with the reference MPRAGE image was found, the SNR and CNR in several regions of interest (ROI) inside the brain were computed for the MPRAGE, three MPRAGE_*like*_ equations, and *syn*MPRAGE for all subjects. The SNR and CNR equations used were the following:

(9) $$SNR_{A}\;=\;\frac{\bar{S_{A}}}{\sigma_{A}},$$

(10) $$CNR_{A-B}\;=\;\frac{\left|\bar{S_{A}}-\bar{S_{B}}\right|}{\sqrt{\sigma_{A}^{2}+\sigma_{B}^{2}}},$$

where $\bar{S_{A}}$ is the mean and $\sigma_{A}$ the standard deviation within ROI (or tissue) A. For this comparison, the reference MPRAGE‐based segmentation was used for all images to avoid effects of segmentation differences.

To correct for remaining signal inhomogeneities in the MPRAGE, MPRAGE_*like*_, and *syn*MPRAGE images, N4 bias field correction^37^, ^38^ was applied as a separate postprocessing steps and the resulting images were included in the further analysis along with the non‐bias‐field corrected images.

To assess the usability of MPRAGE_*like*_ images for neuroimaging applications, the quality of automatic brain segmentation from MPRAGE_*like*_ images was evaluated. Both the N4‐corrected and uncorrected MPRAGE, MPRAGE_*like*_, and *syn*MPRAGE images were segmented using the *FastSurfer* software (v2.3.0) with the “*voxel size independent neural network*” (*FastSurferVINN*)^39^ feature to produce segmentation of brain regions at native input resolution. The N4‐corrected MPRAGE‐based segmentation acted as the reference. The Dice Similarity Coefficient (DSC)^40^, ^41^ and Average Symmetric Surface Distance (ASSD)^42^ metrics were used and computed with the following equations:

(11) $$DSC\;=\;\frac{2\times |G\cup P|}{G+P},$$

where G is the reference segmentation (N4‐corrected MPRAGE), and P, the segmentation from one of the other synthetic T1w images tested in this work, and

(12) $$ASSD\;=\;\frac{\sum_{i=1}^{N_{G}}d_{G\to P,i}+\sum_{i=1}^{N_{P}}d_{P\to G,i}}{N_{G}+N_{P}}.$$

Herein, $d_{G\to P,i}$ is the distance from point i on the surface of the segment from the N4‐corrected MPRAGE to its nearest point on the surface of the segment from one of the other synthetic T1w images tested in this work; $d_{P\to G,i}$ is the distance from point i on the surface of the segment from one of the other synthetic T1w images tested in this work to its nearest point on the surface of the segment from the N4‐corrected MPRAGE; $N_{G}$ and $N_{P}$ are the total number of points on each surface, respectively.

All regions except WM hypointensities (usually negligible in young healthy adults) were used in the calculation of DSC and ASSD. DSC and ASSD values were set to 0 and NaN, respectively for regions where no voxels were included in the given segment.

Finally, the volumes of selected subcortical structures were computed to assess the relative differences in volume against the N4‐corrected MPRAGE (reference) for all three N4‐corrected MPRAGE_*like*_ images. For statistical evaluation of volume differences, a two‐sided Wilcoxon signed rank test^43^ with Bonferroni correction^44^ was applied.

## 3 RESULTS

### 3.1 Optimization of regularization term λ

**FIGURE 2:**
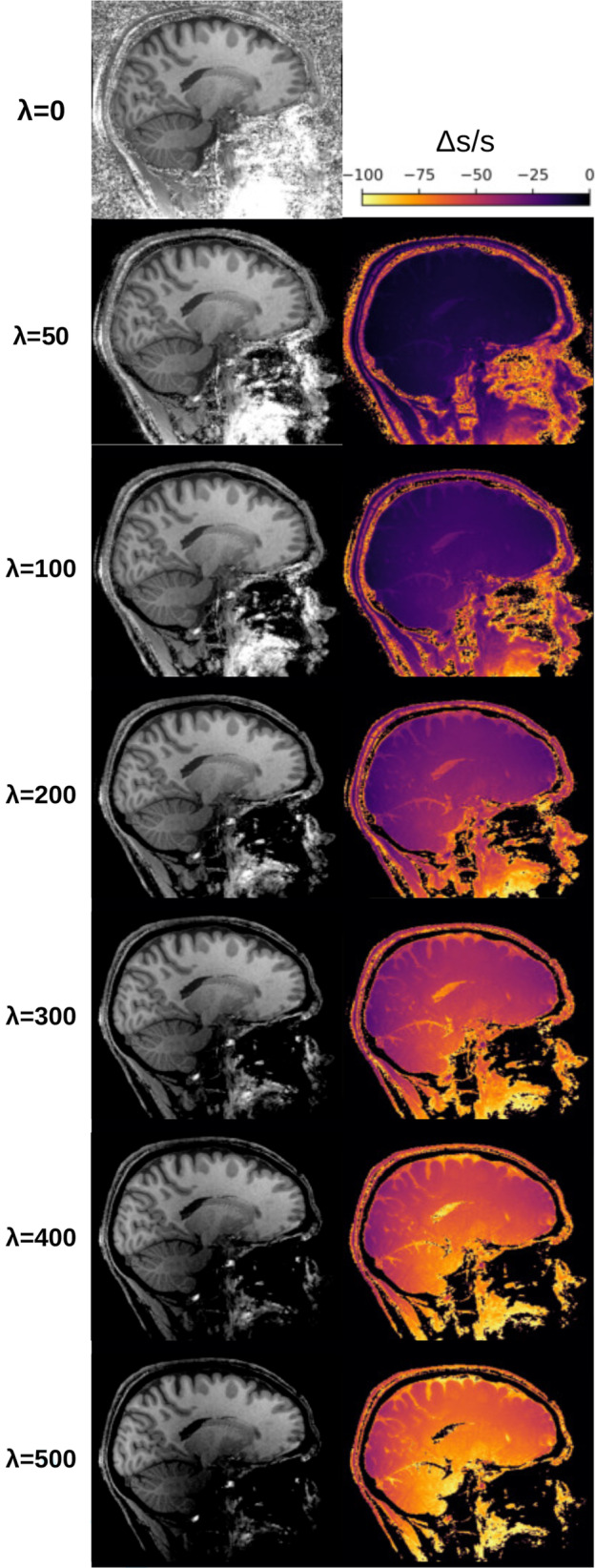
Impact of λ on signal intensity of MPRAGE_*like*_ images for the λ values tested in this study for one example subject. MPRAGE_*like,all*_ images are shown in the left column with their respective Δs/s image on the right (equation shown on the top right). Only the images for Equation (1) are shown due to negligible differences in results between Equations (1), (2), and (3).

The impact of λ on the MPRAGE_*like*_ images is shown in Figure 2. Qualitatively, one can observe that with increasing value of λ, the background noise is reduced while the spatial signal inhomogeneity is increased.

**FIGURE 3:**
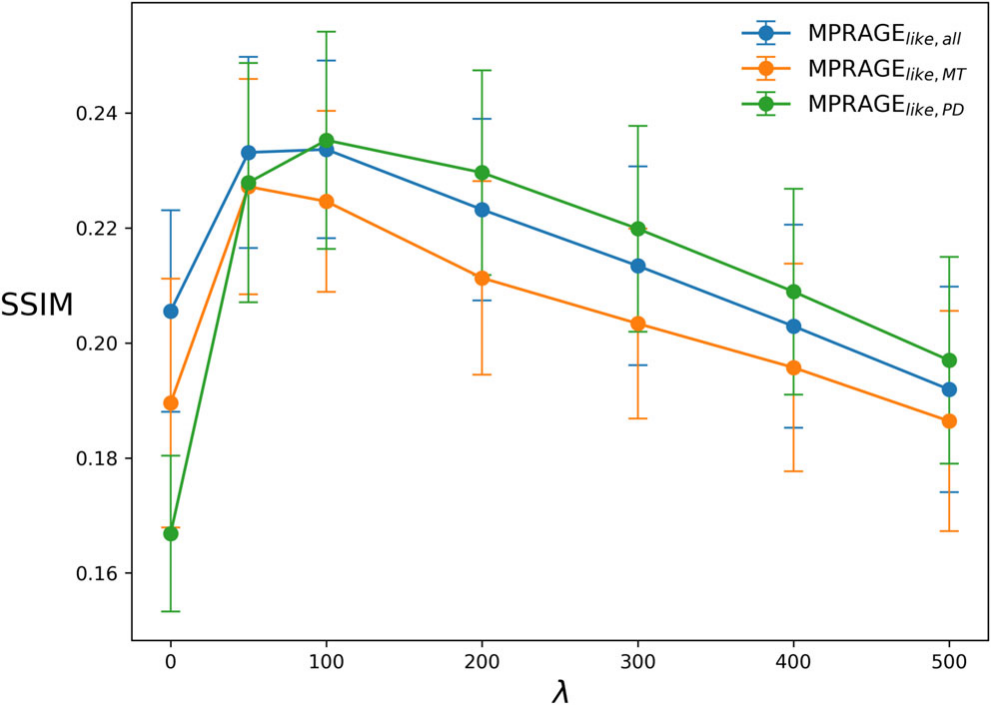
SSIM (maximum: 1.0, minimum: −1.0) computed between the MPRAGE and all λ values tested for the three MPRAGE_*like*_ equations proposed in this work. The dots represent the mean SSIM calculated across all subjects for each λ with the error bars representing the standard deviation.

Quantitative comparisons performed using the average SSIM are shown in Figure 3. The highest SSIM value was reached at λ  = 100 for MPRAGE_*like,all*_ and MPRAGE_*like,PD*_, and λ  = 50 for MPRAGE_*like,MT*_.

Based on these results, λ = 100 was selected as the best regularization term for sufficient background noise suppression while minimally impacting the image contrast.

### 3.2 Qualitative and quantitative comparisons between the acquired MPRAGE, MPRAGE_*like*_ and *syn*MPRAGE

**FIGURE 4:**
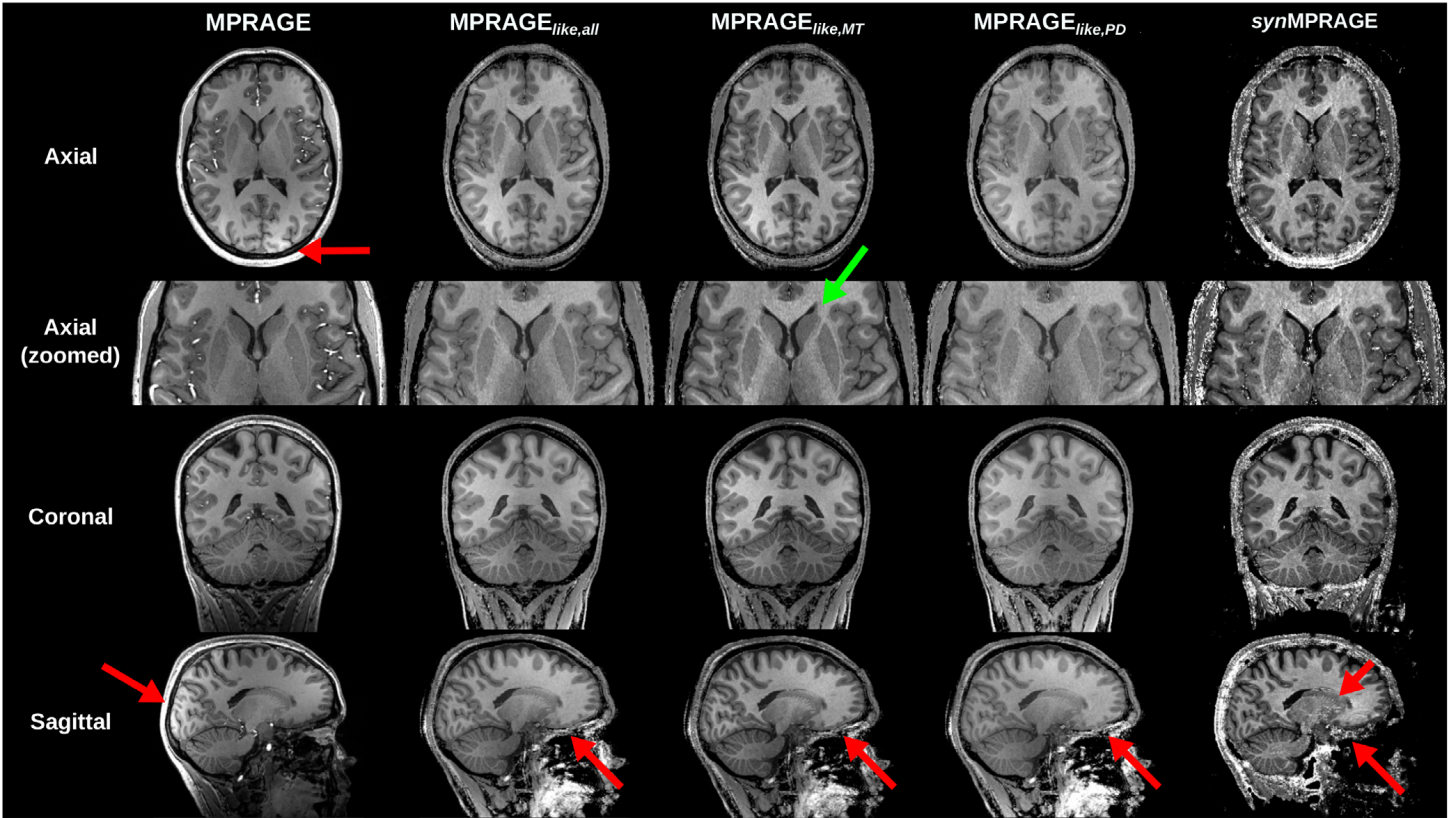
Qualitative comparison between the acquired MPRAGE, three MPRAGE_*like*_, and *syn*MPRAGE (not N4‐corrected) in the three anatomical planes with a zoomed‐in version of the axial plane in the second row for one example subject. Green arrows show well‐defined structures, whereas red arrows show issues in the respective images. The MPRAGE_*like*_ images shown have a λ value of 100.

The acquired MPRAGE, three MPRAGE_*like*_ images (at λ  = 100), and *syn*MPRAGE for one representative subject are shown in Figure 4. All MPRAGE_*like*_ images appeared visually similar to each other and to the acquired MPRAGE. A slight variation in contrast was observed between the three MPRAGE_*like*_ where better overall WM‐GM visual contrast was observed for the MPRAGE_*like,MT*_ compared to the two other MPRAGE_*like*_. One visible difference between MPRAGE_*like*_ images and the MPRAGE was the brightness of blood vessels in the latter compared to the MPRAGE_*like*_. Moreover, receive bias can be observed (red arrows) on the MPRAGE image, which is not present in the other images. The latter, in turn, exhibit different, broad patterns of slight signal inhomogeneities. *syn*MPRAGE showed less‐defined subcortical GM structures compared to MPRAGE_*like*_ and MPRAGE with visibly more noise. MPRAGE_*like*_ and *syn*MPRAGE images exhibited signal brightening in the cortex above the sinuses, which was not observed in the MPRAGE as shown with the red arrows in the bottom row. A significant amount of noise was observed in the skull and skin regions surrounding the brain in the *syn*MPRAGE images compared to the MPRAGE_*like*_ images and MPRAGE.

SNR values are reported in Table 1. Overall, MPRAGE had the highest SNR, the different versions of MPRAGE_*like*_ slightly lower, while *syn*MPRAGE had systematically the lowest SNR for all ROIs.

**TABLE 1:** Mean SNR (± standard deviation) calculated across all subjects for different regions of interest. The non N4‐corrected images were used for the calculations. The largest SNR value between all images for each region is shown in bold.

SNR	MPRAGE	MPRAGE_*like,all*_	MPRAGE_*like,MT*_	MPRAGE_*like,PD*_	*syn*MPRAGE
Cerebral WM	6.7 ± 0.7	**8.0** ± **1.1**	7.2 ± 0.8	8.0 ± 1.2	5.3 ± 0.9
Cerebral cortex	5.5 ± 0.7	5.9 ± 0.7	5.4 ± 0.5	**6.0** ± **0.8**	3.7 ± 0.5
Cerebellum WM	**13.7** ± **2.3**	8.3 ± 1.5	7.5 ± 1.1	8.3 ± 1.5	4.4 ± 1.0
Cerebellum cortex	**6.8** ± **1.3**	5.3 ± 0.7	4.9 ± 0.6	5.5 ± 0.9	2.7 ± 0.4
Putamen	**13.5** ± **1.9**	9.0 ± 1.1	7.9 ± 0.9	9.4 ± 1.1	3.8 ± 0.8
Caudate	**14.0** ± **2.1**	8.8 ± 0.8	7.7 ± 0.7	9.2 ± 0.9	3.1 ± 0.5

Regarding CNR, shown in Table 2, the highest CNR values were obtained by *syn*MPRAGE for the cortex‐WM, whereas MPRAGE_*like,MT*_ had the highest values for the remaining regions of interest.

**TABLE 2:** Mean CNR (± standard deviation) calculated across all subjects for different gray‐white matter adjacent regions. The non N4‐corrected images were used for the calculations. The largest CNR value between all images for each region is shown in bold.

CNR	MPRAGE	MPRAGE_*like,all*_	MPRAGE_*like,MT*_	MPRAGE_*like,PD*_	*syn*MPRAGE
Cortex‐WM	0.89 ± 0.24	1.45 ± 0.22	1.60 ± 0.22	1.20 ± 0.22	**1.71** ± **0.31**
Cerebellum Cortex‐WM	0.94 ± 0.16	1.12 ± 0.33	**1.29** ± **0.35**	0.87 ± 0.29	1.14 ± 0.24
Caudate‐WM	1.76 ± 0.15	1.78 ± 0.26	**1.89** ± **0.23**	1.52 ± 0.27	1.15 ± 0.34
Putamen‐WM	1.56 ± 0.17	1.66 ± 0.21	**1.72** ± **0.22**	1.46 ± 0.22	1.01 ± 0.28

### 3.3 *FastSurferVINN* segmentation results across different MPRAGE‐images

**FIGURE 5:**
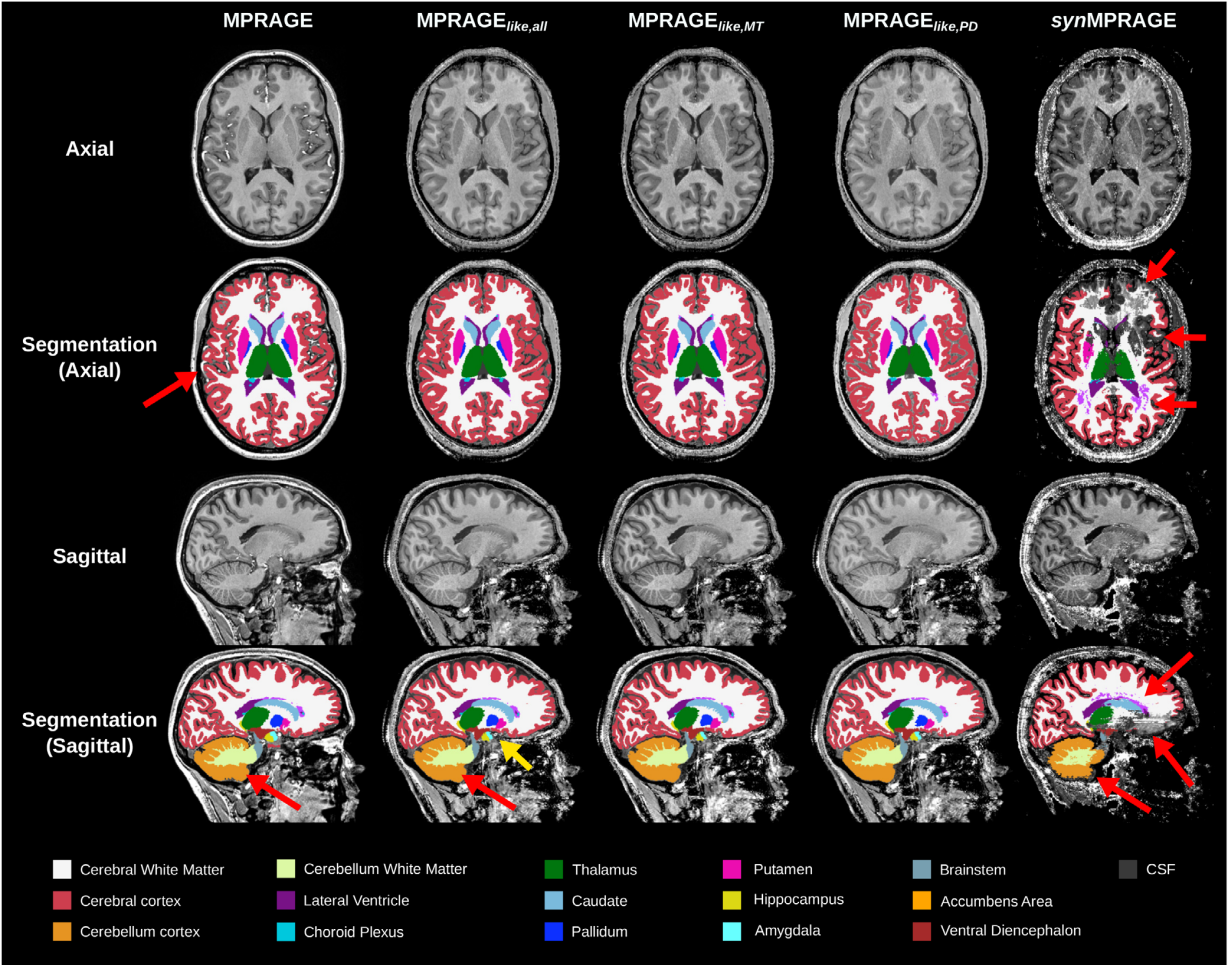
Input images and their corresponding *FastSurferVINN* segmentation results in the axial and sagittal planes for the MPRAGE versus MPRAGE_*like*_ (λ = 100) and *syn*MPRAGE (all N4‐corrected). The cortical region next to the sinuses appears to be challenging for *FastSurferVINN* even for the N4‐corrected MPRAGE where no signal brightening but signal dropouts were observed compared to the MPRAGE_*like*_ and *syn*MPRAGE images. Thin cerebellum WM branches are not fully segmented (red arrows, last row) for all images. Segmentation of subcortical nuclei in the middle brain appears slightly different between the MPRAGE and MPRAGE_*like*_ images (yellow arrow). For *syn*MPRAGE, significant visual differences with MPRAGE_*like*_ are observed. For instance, segmentation in the frontal lobe was difficult, and, in addition, a significant amount of noisy WM regions were segmented as WM‐hypointensities (both identified with red arrows). Several subcortical structures are not correctly segmented compared to the MPRAGE and MPRAGE_*like*_. For example, the caudate, putamen, and pallidum are barely visible on the axial slice, whereas it are clearly visible for MPRAGE and MPRAGE_*like*_. Moreover, a significant portion of the WM and GM over the sinuses is lacking.

The automatic brain segmentation produced by *FastSurferVINN* for N4‐corrected MPRAGE, MPRAGE_*like*_, and *syn*MPRAGE in one example subject are shown in Figure 5. Visually, the MPRAGE_*like*_‐based segmentation appears very similar to the MPRAGE‐based one, while the *syn*MPRAGE‐based segmentation appears different in various regions.

**FIGURE 6:**
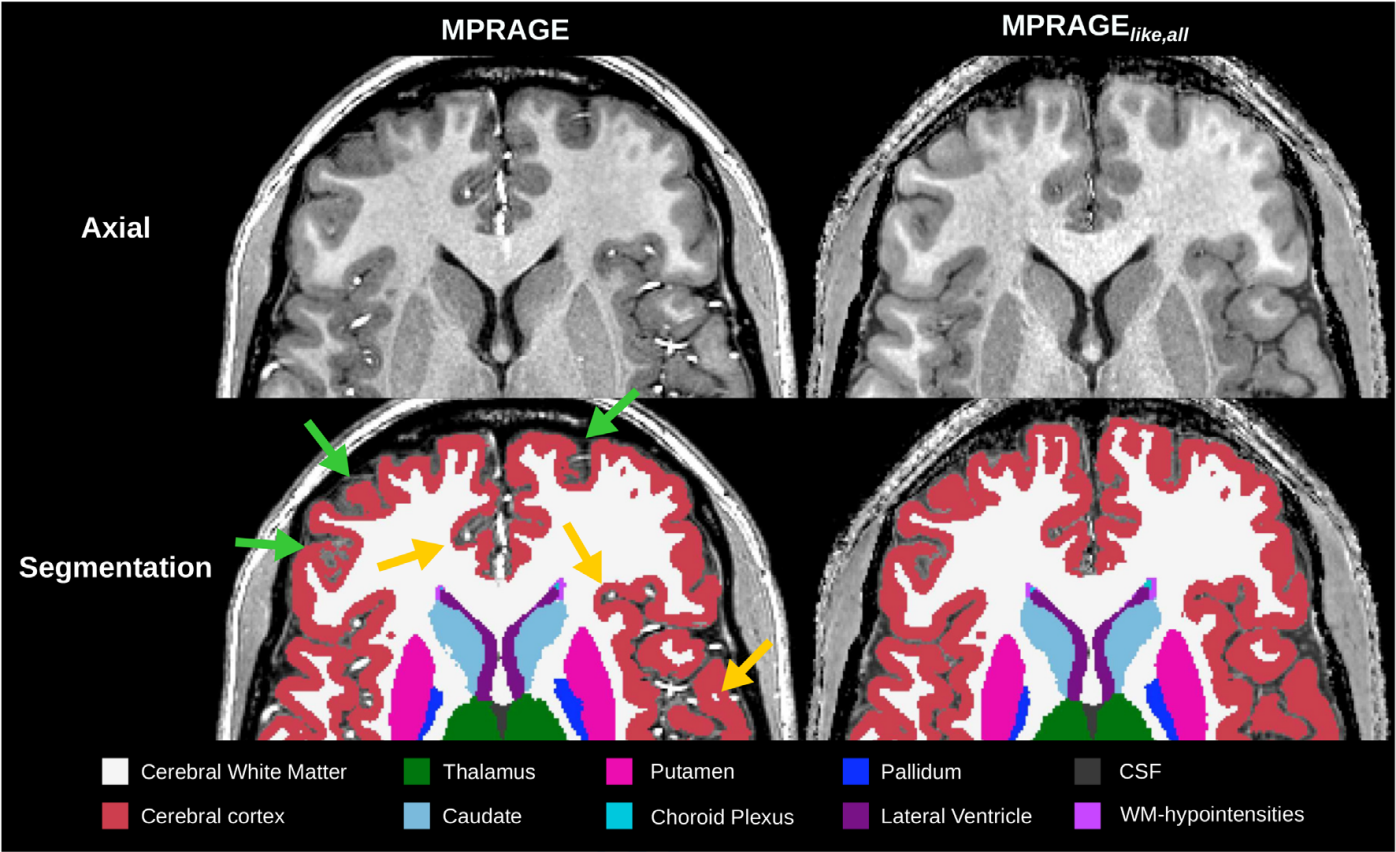
Close‐up view of the N4‐MPRAGE and N4‐MPRAGE_*like,all*_ images in the axial plane (first row) with their corresponding segmentation results (second row). Green arrows represent some discrepancies in the cortex segmentation between the N4‐MPRAGE and N4‐MPRAGE_*like,all*_ and yellow arrows show the impact of bright blood vessels on the cortex segmentation for the N4‐MPRAGE. Structures segmented are shown in the legend at the bottom. Very narrow parts of the lateral ventricles (one voxel width) are depicted on both images. Only the N4‐MPRAGE_*like,all*_ is shown due to negligible differences between MPRAGE_*like*_ segmentations.

To assess the segmentation results in more detail, a close‐up view of the axial plane is shown in Figure 6. Fine structures like small cortical sulci benefit from the (0.6 mm)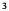 resolution. Overall, the segmentation of subcortical nuclei is very similar between the MPRAGE and MPRAGE_*like,all*_, while differences are observed mainly in the frontal cortex.

**FIGURE 7:**
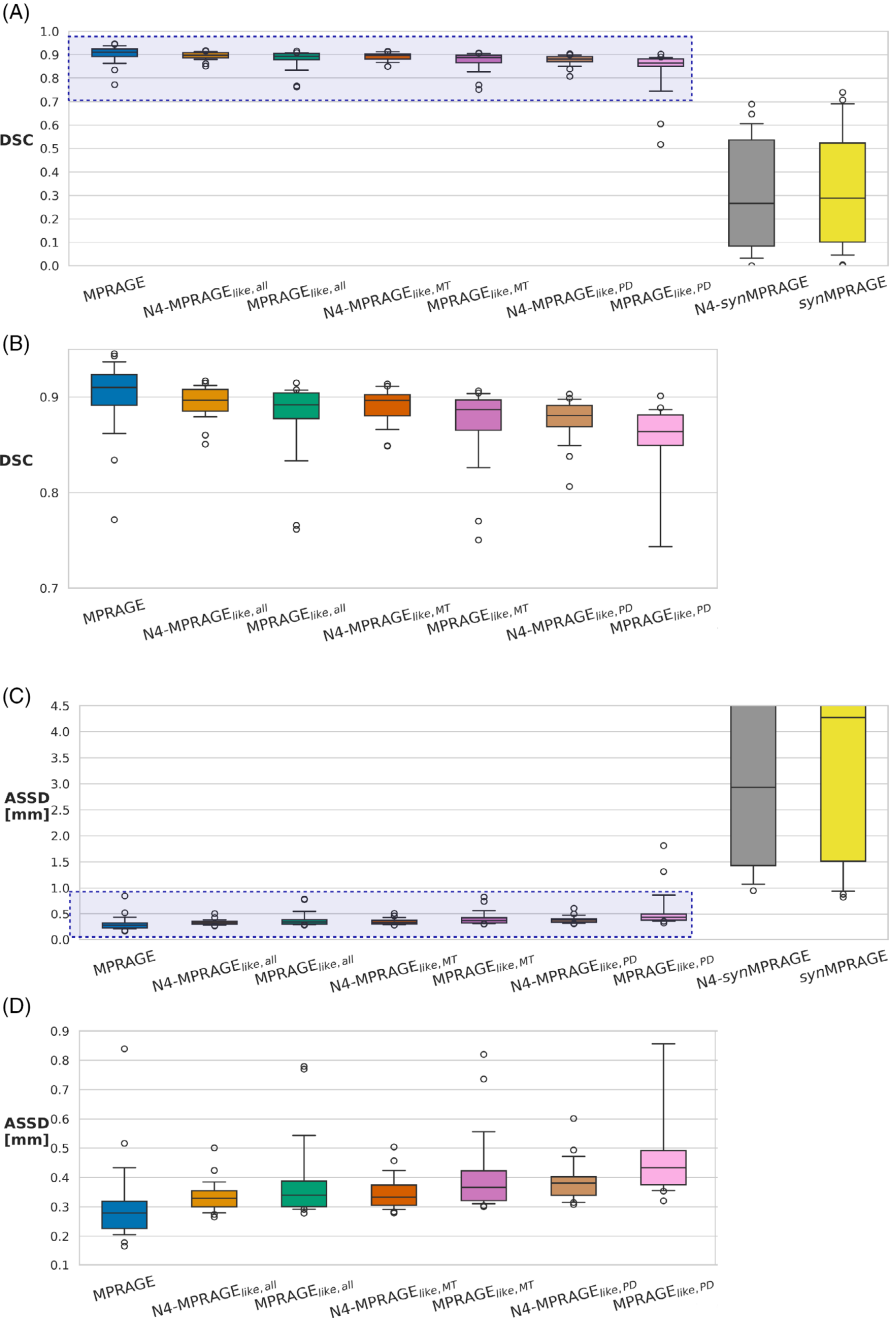
Box plots of (A) the DSC (ideal: 1.0) and (C) ASSD (ideal: 0.0) values calculated across all subjects for every input image tested in this work. A zoomed‐in version (light blue rectangle) of plots (A) and (C) are shown in (B) and (D), respectively. The black line in each box corresponds to the median, the boxes are the interquartile ranges (IQR), the whiskers are the 5th and 95th percentiles, with the circles correspond to outliers.

The average DSC and ASSD calculated against the reference N4‐corrected MPRAGE across all subjects are shown with box plots in Figure 7A,C ((B) and (D) provide a zoom‐in version of (A) and (C), respectively). For both metrics, the non N4‐corrected MPRAGE was the closest match to the reference, with all MPRAGE_*like*_ variations also being highly similar to MPRAGE. All MPRAGE_*like*_ had a median DSC greater than 0.85. Regarding ASSD, all variations of MPRAGE_*like*_ images had a median value below 0.6 mm (i.e., < one voxel). For *syn*MPRAGE, the results were substantially worse; below 0.3 for DSC and greater than 2.5 mm for ASSD for both *syn*MPRAGE versions. One subject was excluded from the DSC and ASSD results due to label errors from *FastSurferVINN* making the output segmentations unusable.

**FIGURE 8:**
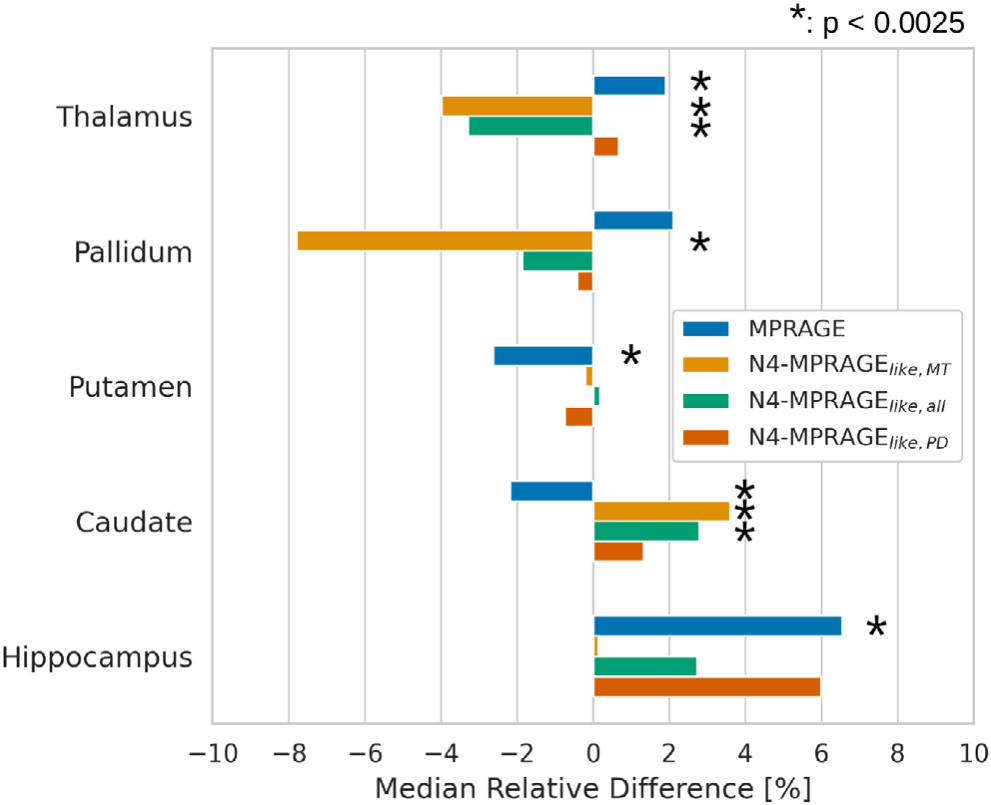
Median relative difference (%) in the volumes calculated across all subjects for the raw MPRAGE and N4‐corrected MPRAGE_*like*_ images tested against the N4‐corrected MPRAGE for selected subcortical GM nuclei. The * represents methods with a P‐value < the Bonferroni corrected significance threshold of 0.0025.

The relative difference in volume for N4‐corrected MPRAGE_*like*_ images compared to the reference N4‐corrected MPRAGE for selected subcortical nuclei are plotted in Figure 8. Most relative differences were below ±5%. The non‐corrected MPRAGE was the technique with the highest number of significant differences in median volumes with 4. N4‐MPRAGE_*like,MT*_ had 3, N4‐MPRAGE_*like,all*_ 2, whereas N4‐MPRAGE_*like,PD*_ had no volume difference that was statistically significant for all ROIs tested.

## 4 DISCUSSION

This study explores the feasibility of synthesizing images of pure T1w contrast directly from MPM or VFA source images without the requirement to calculate quantitative parameters maps beforehand. The proposed MPRAGE_*like*_ images provide a robust surrogate to acquired MPRAGE for automatic brain segmentation with sub‐millimeter resolution at 7T. All MPRAGE_*like*_ equations with λ = 100 have shown to be more robust and high‐quality substitutes to MPRAGE than the previously proposed *syn*MPRAGE.

### 4.1 Optimization of regularization term λ

Selecting the optimal λ depends on the overall scaling of image intensities and is a trade‐off between removing the background noise and preserving signal homogeneity across the brain, primarily in the cerebellum and temporal lobes, as also reported in Reference 9. In this work, a λ value of 100 was adopted due to the fact that (1) the vast majority of the background noise was removed, (2) there was minimal signal inhomogeneity reintroduced in the brain, and (3) two of the three MPRAGE_*like*_ equations achieved their highest SSIM value at that λ. For MPRAGE_*like,MT*_, λ = 100 was the second largest SSIM, which was very close to the value at λ = 50. Whole‐brain image intensities at the first TE of MTw, T1w, and PDw data in this study roughly ranged between 500 and 2500. For differently acquired/scaled images, correspondingly scaled λ values are expected to perform comparably.

The SSIM values reported in Figure 3 are arguably low compared to other techniques to produce synthetic MPRAGE images.^22^ SSIM is often used in contexts where one wants to evaluate the impact of a “lossy” compression process on an uncompressed image or the quality of an image restoration process.^45^ However, in this work, the SSIM was computed against the acquired MPRAGE, which was a different image altogether, acquired independently from the MPM images. Furthermore, the entire field of view was evaluated, which is why a large fraction of voxels outside the brain presumably dominate the SSIM metric. Considering this, the low SSIM values obtained are not surprising nor concerning. SSIM was chosen because a metric that would convey a sense of perceived similarity over a pure quantitative similarity was desired, especially since the voxel values of an MPRAGE image do not convey any direct quantitative meaning.

### 4.2 Qualitative and quantitative comparisons between the acquired MPRAGE, MPRAGE_*like*_, and *syn*MPRAGE

Qualitatively, all MPRAGE_*like*_ images looked quite similar to each other. The added MTw contrast, both for the MPRAGE_*like,all*_ and MPRAGE_*like,MT*_, helped improve the visual contrast between subcortical nuclei and WM compared to the MPRAGE_*like,PD*_. The *syn*MPRAGE image posed further challenges. As seen from Figure 4, the noisy background of *syn*MPRAGE images was not possible to mask completely. Several combinations of masking techniques as removing outliers and negative values for quantitative parameters were tested in this work but none ended up efficiently masking the background noise without removing relevant anatomy inside the brain. Also, no simple regularization approach can be applied on the *syn*MPRAGE images as done for MPRAGE_*like*_. Consequently, the remaining noisy regions next to CSF around the brain made it difficult to properly detect borders of structures as seen with the segmentation results (Figure 5). It is noteworthy that R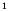 mapping at high resolution in very limited scan time is very challenging, and more sophisticated fitting algorithms compared to the MPM equations, such as Bayesian fitting or other approaches, could potentially have reduced the noise level in the R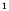 maps and thereby produced higher quality *syn*MPRAGE images.^21^

The SNR analysis did not show any large difference between the three proposed MPRAGE_*like*_ equations. Overall, both the MPRAGE_*like,all*_ and MPRAGE_*like,PD*_ had similar SNR values, which were always higher than the values reported for MPRAGE_*like,MT*_. Additionally, all MPRAGE_*like*_ images had higher SNR than the *syn*MPRAGE. For the putamen, caudate, and cerebellar cortex, the SNR values of MPRAGE_*like*_ images reached around a factor of two higher than those of *syn*MPRAGE. This may largely be due to R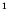 estimation outliers that increased the signal standard deviation in the selected ROIs. Considering the MPRAGE_*like,all*_ equation includes three contrasts (instead of two for MPRAGE_*like,PD*_ and MPRAGE_*like,MT*_), an SNR gain could have been expected, but the lower signal in MTw images used for MPRAGE_*like,MT*_ resulted in no observable SNR gain for MPRAGE_*like,all*_. Conversely, the increased contrast of MTw images improved the CNR values for MPRAGE_*like,all*_.

While *syn*MPRAGE showed the highest WM‐GM CNR (driven by particularly high WM‐GM signal difference in the numerator that seemed to outweigh the large noise standard deviation in the denominator of Equation (10)), the low CNR values (and SNR) in the subcortical structures caused problems for *FastSurferVINN* to properly segment the *syn*MPRAGE images. As a result, at least one region was missing completely in the segmentations for 21 of the 23 subjects, which explains the large difference in DSC and ASSD values compared to the different MPRAGE_*like*_ images. This makes the segmentation results unfit for further use. For comparison, no region was missing for MPRAGE_*like,all*_ and MPRAGE_*like,MT*_ across all subjects, whereas only one region in one subject was missing for MPRAGE_*like,PD*_.

All MPRAGE_*like*_ equations produced highly comparable image quality while all of them being superior to the *syn*MPRAGE in every aspect except CNR between WM‐GM. Ultimately, the fact that all MPRAGE_*like*_ equations produced highly comparable images gives flexibility to researchers to calculate a MPRAGE_*like*_ image based on the contrasts available in their MPM/VFA protocols. For instance, acquiring only T1w and PDw contrasts for R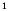 estimation using the VFA method allows one to compute MPRAGE_*like,PD*_ images similar to the ones presented here. This should even work in short scan time using single‐TE VFA acquisitions as shown with Figure S1 in the Supporting Information.

One advantage of MPRAGE_*like*_ is its simplicity. Indeed, neither fitting of quantitative parameters nor DL training are required like in comparable studies.^21^, ^22^, ^24^ However, since an R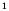 map is commonly computed in neuroimaging pipelines, *syn*MPRAGE is also a straightforward technique to implement, which motivated the choice for comparison against MPRAGE_*like*_.

### 4.3 Automatic brain segmentation from *FastSurferVINN* versus other segmentation techniques

Automatic brain segmentation algorithms optimized for 7T images are limited. To the best of our knowledge, this study is the first one using *FastSurferVINN* for 7T images at 0.6 mm isotropic nominal resolution. Indeed, *FastSurferVINN* was designed for T1w MPRAGE images at ≤ 3T with resolutions between (0.7 and 1.0 mm)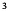, meaning that the (0.6 mm)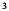 resolution used in this work was considered an *“experimental resolution”* by *FastSurferVINN*. However, previous analyses done with *FastSurferVINN* showed its robustness to higher resolutions unseen from the training dataset.^39^ From Figure 6, we could observe that *FastSurferVINN* handled well the (0.6 mm)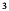 resolution of the images. The high resolution enabled proper segmentation of thin cortical regions, which would not have been possible at 1 mm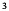.

It is worth noting that universal pTx pulses were used, and therefore, the level of $B_{1}^{+}$ inhomogeneities observed in the images was considerably lower than what is typically observed for 7T images using circular‐polarized (CP) excitation only (See Figures S2 and S3 from Supporting Information). Indeed, flip angles between 75% and 125% of the nominal flip angle value for UP excitation were observed across the brain, which corresponds to a substantial improvement over CP excitation, where values as low as 25% were observed (Figure S3). Ultimately, that resulted in the N4‐correction being effective to correct for the remaining receive inhomogeneities in a similar fashion as it would at 3T.

In a pilot study,^46^ several alternative segmentation algorithms were considered and evaluated. These included *FreeSurfer*, *SynthSeg* ^47^ and *CEREBRUM‐7T*.^48^ For *FreeSurfer*, a specific pipeline for sub‐millimeter 7T images has been previously proposed in the literature.^49^ However, in our pilot study, the *FreeSurfer* pipeline showed a high sensitivity to $B_{1}^{+}$ inhomogeneities in addition to a limited capacity in handling sub‐millimeter resolution. Moreover, published studies about neurological diseases (e.g., Alzheimer's disease) have shown that *FastSurferVINN* gives significantly better or equivalent results to *FreeSurfer* while also being, on average, 40 times faster.^50^ *SynthSeg* was excluded from this work due to its limitation in output resolution of only 1 mm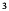, while *CEREBRUM‐7T* required MP2RAGE as the input image and produces a limited number of segmented regions. Hence, *FastSurferVINN* was selected as the segmentation method in this study, with the N4‐corrected MPRAGE images used as reference.

### 4.4 *FastSurferVINN* segmentation results across different MPRAGE‐images

In this work, we found that any MPRAGE_*like*_ image provided a more robust and reliable input to automatic brain segmentation than *syn*MPRAGE. The segmentation results from MPRAGE_*like*_ images were highly comparable to those obtained from the reference MPRAGE. Limitations observed in the MPRAGE_*like*_‐based segmentations, such as coarse delineations of fine WM‐GM boundaries in the cerebellum and the cortical region over the sinuses, were also observed in the MPRAGE‐based segmentations. This lets us believe that these limitations are not due to the image quality of MPRAGE_*like*_, but from the segmentation algorithm used. Indeed, thin WM branches like the ones observed in the cerebellum are often problematic to segment for DL‐based techniques since very few high‐resolution segmented datasets are available for the training dataset. Even datasets like the Human Connectome Project (HCP),^51^ which includes (0.7 mm)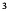 segmentations from 3T MPRAGE images, do not have well‐segmented cerebral WM branches due to the limited capacity of *FreeSurfer* (software used for the HCP) to segment sub‐millimeter images and limited cerebral WM‐GM contrast at 3T.

The high variability in image quality for *syn*MPRAGE between subjects was due to the estimation process of the R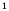 maps, which negatively affected the overall segmentation quality. Quantitatively, that could be observed with the IQRs of the DSC values computed. Indeed, some subjects had high‐quality *syn*MPRAGE images, which resulted in average DSC values around 0.70, but these represent outliers in the distribution of subjects. Reliable image quality is an important factor, and MPRAGE_*like*_ showed a considerably superior robustness and reliability in comparison to *syn*MPRAGE.

The fact that MPRAGE_*like*_ segmentation showed only minor improvement after the N4‐correction may be due to the low λ value used, such that receive inhomogeneities are by design almost completely canceled due to the division of two images with identical receive bias in a very similar fashion as MP2RAGE.^8^ For *syn*MPRAGE, the observed decrease in DSC and increase in ASSD after N4‐correction might be due to the fact that *syn*MPRAGE images do not exhibit receive inhomogeneities as expected by the N4 algorithm, potentially resulting in suboptimal effects.

Even if MPRAGE_*like,MT*_ gave the lowest SNR and SSIM values among MPRAGE_*like*_ images, the output segmentations were not affected. This lets us believe that the superior WM‐GM contrast of MPRAGE_*like,MT*_ is important for its segmentability. On the other hand, MPRAGE_*like,PD*_, which had the highest SNR and SSIM, both uncorrected and N4‐corrected images produced the lowest median DSC and ASSD among all MPRAGE_*like*_ images. MPRAGE_*like,all*_ always held the highest DSC and ASSD values (by a very small margin) while never having the highest SNR, CNR, nor SSIM. In the end, the segmentation results of MPRAGE_*like*_ were highly similar with negligible differences between them, suggesting that (1) the added MTw contrast helps for segmentation and (2) that any of them can be used as a great surrogate to MPRAGE for T1w‐based segmentation algorithms.

The DSC values found in this work for the MPRAGE_*like*_ are very similar to other values reported in the literature.^22^ In Reference 22, an average DSC of 0.88 ± 0.02 was reported across subjects. Here, the DSC (and ASSD) were computed for all segmented regions except the WM‐hypointensities (i.e., 34 regions in total) for each subject, whereas only seven selected regions were included in the DSC calculation in Reference 22. If only the same seven regions were included, average DSC of 0.93 ± 0.01, 0.92 ± 0.01, and 0.92 ± 0.02 would have been obtained here for N4‐corrected MPRAGE_*like,all*_, MPRAGE_*like,MT*_, and MPRAGE_*like,PD*_, respectively.

Most of the median relative differences in volume values calculated had statistically significant differences for the non‐corrected MPRAGE. This highlights the relevance of the N4‐correction when volumetric analysis is performed. Moreover, it is worth mentioning that even if N4‐MPRAGE_*like,PD*_ had the lowest DSC and highest ASSD values among MPRAGE_*like*_ images, it was the only one without a significant difference with the reference.

Ultimately, the new approach proposed in this work is a simple and flexible technique to synthesize T1w images directly from multi‐contrast spoiled GRE images. In addition to serving as an anatomical reference image, MPRAGE_*like*_ images can serve as a reliable surrogate for automated brain segmentation if MPM or VFA data is acquired as part of a long neuroscience imaging protocol with tight time constraints.

### 4.5 Limitations

All data presented in this work were acquired at 7T, and both the MPM and MPRAGE protocols were based on non‐product sequences using Universal Pulses pTx. Extrapolation of our findings to non‐pTx MPM images at 7T or 3T is therefore limited. While not investigated in this study, previous works suggested that circularly polarized excitation for MPM at 7T resulted in poor MPRAGE_*like*_ contrast in regions sensitive to $B_{1}^{+}$ inhomogeneities, essentially due to insufficient T1w contrast in the numerator of the MPRAGE_*like*_ equation.^52^ This cannot be compensated by N4‐correction (Supporting Information Figure S2). Moreover, the MPM protocol used was designed to be as fast as possible, which might have resulted in suboptimal contrasts. While MPM protocols are used both at 3T and 7T, the new MPRAGE_*like*_ technique has neither been thoroughly tested at 3T nor with single‐TE data. However, single‐subject data shown in Supporting Information Figure S1 demonstrates that the method can, in principle, be applied at 3T and even using single‐TE data as well. The MPRAGE_*like*_ approach should be further tested on other data acquired on lower field strengths and at other sites with different sequence implementations.

Moreover, it is also important to mention that all subjects in this study were healthy, young, and able to stay still for long periods of time. The MPM sequence used in this study is intended to be used in imaging protocols characterizing neurodegenerative diseases. Even though each of the three MPM contrast acquisitions had shorter scan times at (0.6 mm)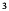 voxel size than the reference MPRAGE acquisition at (0.6 mm)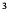 voxel size (TA = 6:00/4:05/4:05 min vs. TA=7:24 min) and inter‐volume motion is accounted for by retrospective registration, intra‐volume motion artifacts may become limiting in patients with symptoms, where excessive motion throughout all MPM scans might result in lower image quality for MPRAGE_*like*_ images.

The segmentations produced from the N4‐corrected MPRAGE images were considered as the reference in this work to compare MPRAGE_*like*_ and *syn*MPRAGE segmentations. However, this is considered a “silver standard” since manual segmentations are typically considered the gold standard. Producing manual segmentation of several regions of the complete brain for 23 subjects at 0.6 mm isotropic resolution is a considerable challenge that requires a substantial amount of time and expertise, which was beyond the scope of this study.

## 5 CONCLUSIONS

In summary, we propose MPRAGE_*like*_; a novel and simple technique producing images with synthetic T1w contrast directly from ME‐GRE images with different contrast weightings that can be used as a surrogate to MPRAGE for automatic brain segmentation. The results showed that MPRAGE_*like*_ produces images highly resembling MPRAGE images except for the absent arterial blood highlighting typical at 7T. Additionally, employing MPRAGE_*like*_ instead of *syn*MPRAGE substantially improves image quality and segmentation results. This makes MPRAGE_*like*_ a superior alternative to *syn*MPRAGE and offers a robust new solution for synthetic T1w imaging techniques at UHF‐MRI, which has been previously lacking. MPRAGE_*like*_ would benefit from further validation across different field strengths and within non‐healthy cohorts. Ultimately, implementing MPRAGE_*like*_ would allow researchers to skip acquiring a MPRAGE resulting in a considerable amount of scan time saved while still being able to run the same quantitative neuroimaging pipelines.

## CONFLICT OF INTEREST STATEMENT

The authors declare no potential conflict of interests.

## Supporting information




**Data S1.** Supporting Information.

## Data Availability

A python implementation of the proposed method can be found at https://github.com/mafortin/mprage‐like.
