# Supplementary material for: MPRAGE like : A novel approach to generate T1w images from multi‐contrast gradient echo images for brain segmentation
Source: Magn Reson Med. 2025 Feb 4;94(1):134–49. doi: 10.1002/mrm.30453 (PMC12021339; doi:10.1002/mrm.30453)
Supplement: Supplementary file 1 — Data S1. Supporting Information. [file MRM-94-134-s001.pdf]

MPRAGE$like$: a novel approach to generate T1w images from Multi-Contrast Gradient Echo images for brain segmentation: Supplementary Information

1. DERIVATIONS OF MPRAGE$_{like}$ SIGNAL EQUATIONS BASED ON MPM THEORY

In this section, the derivations of Eqs. 4, 5 and 6 presented in section 2.4 of the paper are shown based on the MPM signal equations as presented in Refs. [1, 2].

From Eq. 7 in Ref. [1], we can obtain an approximation of the signal equation for the T1w and PDw MPM images:

$$S \cong A\alpha \frac{\mathrm{TR} \cdot R_1}{\alpha^2/2 + \mathrm{TR} \cdot R_1} \tag{S1}$$

where A is the amplitude of the spoiled GRE at the echo time, TR, the repetition time, α, the apparent flip angle and, R_1, the longitudinal relaxation rate. Additionally, we can replace some terms of Eq. S1 by the following equations:

$$\alpha = f_\mathrm{T}\alpha_\mathrm{nom} \tag{S2}$$

$$A = \mathrm{PD} f_\mathrm{R} \tag{S3}$$

$$R_1 = R_{1\mathrm{app}} f_\mathrm{T}^2 \tag{S4}$$

where α_nom is the nominal flip angle, f_T, the multiplicative bias transmit correction factor, f_R, the multiplicative bias receive correction factor, PD, a scaling factor proportional to the proton density and, $R_{1\mathrm{app}}$, the apparent longitudinal relaxation rate. Thus, we obtain the two following approximations for the T1w and PDw signal respectively:

$$S_\mathrm{T1} \cong \mathrm{PD} f_\mathrm{R} f_\mathrm{T} \frac{\alpha_{\mathrm{nom}_\mathrm{T1}} \cdot \mathrm{TR}_\mathrm{T1} \cdot R_{1\mathrm{app}}}{\alpha^2_{\mathrm{nom}_\mathrm{T1}}/2 + \mathrm{TR}_\mathrm{T1} \cdot R_{1\mathrm{app}}} \tag{S5}$$

$$S_\mathrm{PD} \cong \mathrm{PD} f_\mathrm{R} f_\mathrm{T} \frac{\alpha_{\mathrm{nom}_\mathrm{PD}} \cdot \mathrm{TR}_\mathrm{PD} \cdot R_{1\mathrm{app}}}{\alpha^2_{\mathrm{nom}_\mathrm{PD}}/2 + \mathrm{TR}_\mathrm{PD} \cdot R_{1\mathrm{app}}} \tag{S6}$$

For the MTw MPM image, we can use Eqs. 6 and 10 in Ref. [2] and we obtain:

$$S_\mathrm{MT} \cong \mathrm{PD} f_\mathrm{R} f_\mathrm{T} \frac{\alpha_{\mathrm{nom}_\mathrm{MT}} \cdot \mathrm{TR}_\mathrm{MT} \cdot R_{1\mathrm{app}}}{\alpha^2_{\mathrm{nom}_\mathrm{MT}}/2 + \delta_\mathrm{app} + \mathrm{TR}_\mathrm{MT} \cdot R_{1\mathrm{app}}} \tag{S7}$$

where δ_app is the apparent MT saturation.

With Eqs. S5 and S6, it is possible to derive Eq. 6 from Eq. 3 with λ=0 such that

$$MPRAGE_{like,PD} \cong \frac{S_\mathrm{T1}}{S_\mathrm{PD}} \tag{S8}$$

but here TR_T1 = TR_PD = TR. Therefore, we have the following equation:

$$MPRAGE_{like,PD} \cong \frac{\mathrm{PD} f_\mathrm{R} f_\mathrm{T} \frac{\alpha_{\mathrm{nom}_\mathrm{T1}} \cdot \mathrm{TR} \cdot R_{1\mathrm{app}}}{\alpha^2_{\mathrm{nom}_\mathrm{T1}}/2 + \mathrm{TR} \cdot R_{1\mathrm{app}}}}{\mathrm{PD} f_\mathrm{R} f_\mathrm{T} \frac{\alpha_{\mathrm{nom}_\mathrm{PD}} \cdot \mathrm{TR} \cdot R_{1\mathrm{app}}}{\alpha^2_{\mathrm{nom}_\mathrm{PD}}/2 + \mathrm{TR} \cdot R_{1\mathrm{app}}}} \tag{S9}$$

where all the common terms cancel each other, giving the following result:

$$MPRAGE_{like,PD} \cong \frac{\frac{\alpha_{\mathrm{nom}_\mathrm{T1}}}{\alpha^2_{\mathrm{nom}_\mathrm{T1}}/2 + \mathrm{TR} \cdot R_{1\mathrm{app}}}}{\frac{\alpha_{\mathrm{nom}_\mathrm{PD}}}{\alpha^2_{\mathrm{nom}_\mathrm{PD}}/2 + \mathrm{TR} \cdot R_{1\mathrm{app}}}} \tag{S10}$$

which can be further simplified for better readability to the following:

$$MPRAGE_{like,PD} \cong \left(\frac{\alpha_{\text{nom}_{\text{T1}}}}{\alpha_{\text{nom}_{\text{PD}}}}\right) \cdot \left(\frac{\alpha^2_{\text{nom}_{\text{PD}}} \; + \; 2 \cdot \text{TR} \cdot R_{1\text{app}}}{\alpha^2_{\text{nom}_{\text{T1}}} \; + \; 2 \cdot \text{TR} \cdot R_{1\text{app}}}\right) \tag{S11}$$

where the remaining dependencies on nominal flip angle and TR values can be observed and where we can also notice that the PD and both receive and transmit correction factors are canceled by combining the images†.

For Eq. 5, we have:

$$MPRAGE_{like,MT} \cong \frac{S_{\text{T1}}}{S_{\text{MT}}} \tag{S12}$$

that can be reformulated into:

$$MPRAGE_{like,MT} \cong \frac{\text{PD} f_{\text{R}} f_{\text{T}} \frac{\alpha_{\text{nom}_{\text{T1}}} \cdot \text{TR}_{\text{T1}} \cdot R_{1\text{app}}}{\alpha^2_{\text{nom}_{\text{T1}}}/2 + \text{TR}_{\text{T1}} \cdot R_{1\text{app}}}}{\text{PD} f_{\text{R}} f_{\text{T}} \frac{\alpha_{\text{nom}_{\text{MT}}} \cdot \text{TR}_{\text{MT}} \cdot R_{1\text{app}}}{\alpha^2_{\text{nom}_{\text{MT}}}/2 + \delta_{\text{app}} + \text{TR}_{\text{MT}} \cdot R_{1\text{app}}}} \tag{S13}$$

and further simplified into the following result:

$$MPRAGE_{like,MT} \cong \left(\frac{\alpha_{\text{nom}_{\text{T1}}} \text{TR}_{\text{T1}}}{\alpha_{\text{nom}_{\text{MT}}} \text{TR}_{\text{MT}}}\right) \cdot \left(\frac{\alpha^2_{\text{nom}_{\text{MT}}} + 2 \cdot (\delta_{\text{app}} + \text{TR}_{\text{MT}} \cdot R_{1\text{app}})}{\alpha^2_{\text{nom}_{\text{T1}}} + 2 \cdot \text{TR}_{\text{T1}} \cdot R_{1\text{app}}}\right) \tag{S14}$$

Finally, it is possible to derive Eq. 4 from Eq. 1 with *lambda*=0 with Eqs. S5, S6 and S7, such that

$$MPRAGE_{like,all} \cong \frac{S_{\text{T1}}}{0.5 \times (S_{\text{MT}} \; + \; S_{\text{PD}})} \tag{S15}$$

Consequently, we obtain the following equation:

$$MPRAGE_{like,all} \cong \tag{S16}$$

$$\frac{\text{PD} f_{\text{R}} f_{\text{T}} R_{1\text{app}}}{0.5 \cdot \text{PD} f_{\text{R}} f_{\text{T}} R_{1\text{app}}} \times \frac{\frac{\alpha_{\text{nom}_{\text{T1}}} \text{TR}_{\text{T1}}}{\alpha^2_{\text{nom}_{\text{T1}}}/2 + \text{TR}_{\text{T1}} \cdot R_{1\text{app}}}}{\left(\frac{\alpha_{\text{nom}_{\text{MT}}} \text{TR}_{\text{MT}}}{\alpha^2_{\text{nom}_{\text{MT}}}/2 + \delta_{\text{app}} + \text{TR}_{\text{MT}} \cdot R_{1\text{app}}} + \frac{\alpha_{\text{nom}_{\text{PD}}} \text{TR}_{\text{PD}}}{\alpha^2_{\text{nom}_{\text{PD}}}/2 + \text{TR}_{\text{PD}} \cdot R_{1\text{app}}}\right)}$$

where the equation can be simplified and reorganized for better readability as follows:

$$MPRAGE_{like,all} \cong \frac{\frac{\alpha_{\text{nom}_{\text{T1}}} \text{TR}_{\text{T1}}}{\alpha^2_{\text{nom}_{\text{T1}}} + 2 \cdot \text{TR}_{\text{T1}} \cdot R_{1\text{app}}}}{0.5 \times \left(\frac{\alpha_{\text{nom}_{\text{MT}}} \text{TR}_{\text{MT}}}{\alpha^2_{\text{nom}_{\text{MT}}} + 2 \cdot (\delta_{\text{app}} + \text{TR}_{\text{MT}} \cdot R_{1\text{app}})} + \frac{\alpha_{\text{nom}_{\text{PD}}} \text{TR}_{\text{PD}}}{\alpha^2_{\text{nom}_{\text{PD}}} + 2 \cdot \text{TR}_{\text{PD}} \cdot R_{1\text{app}}}\right)} \tag{S17}$$

† Ultimately, it is important to mention that transmit inhomogeneities are still represented by $R_{1\text{app}}$ and δ_{app} in the final MPRAGE$_{like}$ equations since only the first-order terms are included in these signal approximations. Thus, remaining inhomogeneities of higher order terms are still present as explained in Ref. [3].

2. SUPPLEMENTARY FIGURES

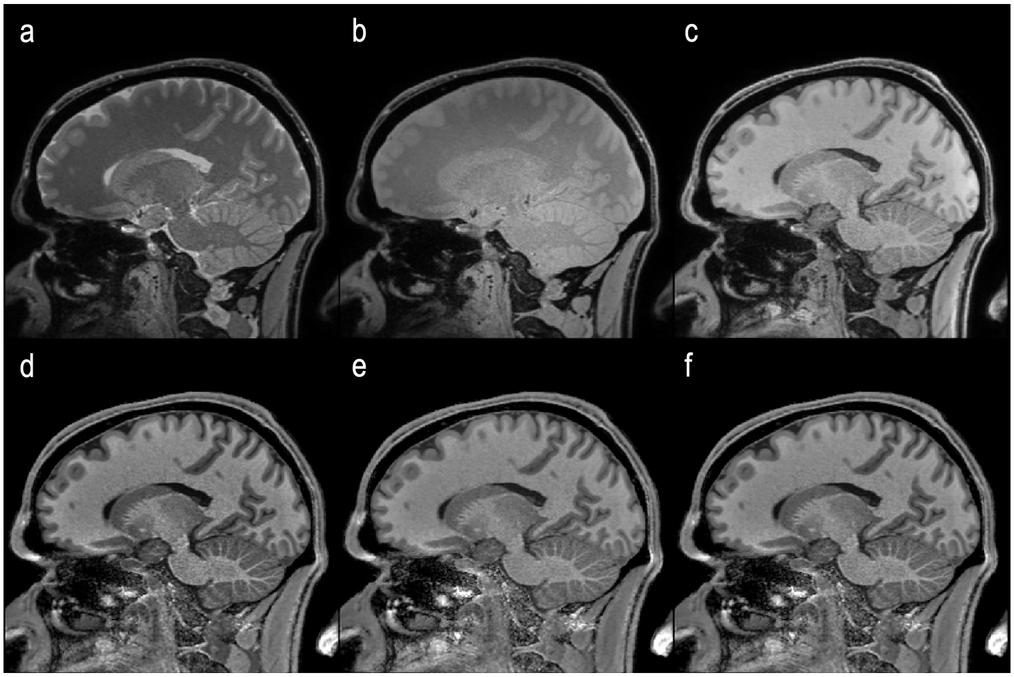

Fig. S1. Sagittal views of whole-head single-TE (TE=6 ms) MTw (a, TR=29 ms/FA=6°), PDw (b, TR=16 ms/FA=4°) and T1w (c, TR=16 ms/FA=23°) MPM data acquired on a 3T (MAGNETOM Prisma) scanner at 0.8 mm isotropic resolution within TA=6:34 min using the same skipped-CAIPI 3D-EPI sequence as used in this work. The bottom row shows different MPRAGE*like* variations after N4 correction using λ=100: MPRAGE*like*, *MT* (d), MPRAGE*like*, *PD* (e) and MPRAGE*like*, *all* (f). This demonstrates that the proposed method can potentially be applied in vastly different imaging domains (e.g. 3T, single-TE, body-coil CP-mode excitation) as the one investigated in the current work (7T, multi-TE, 8-channel-Tx UP excitation).

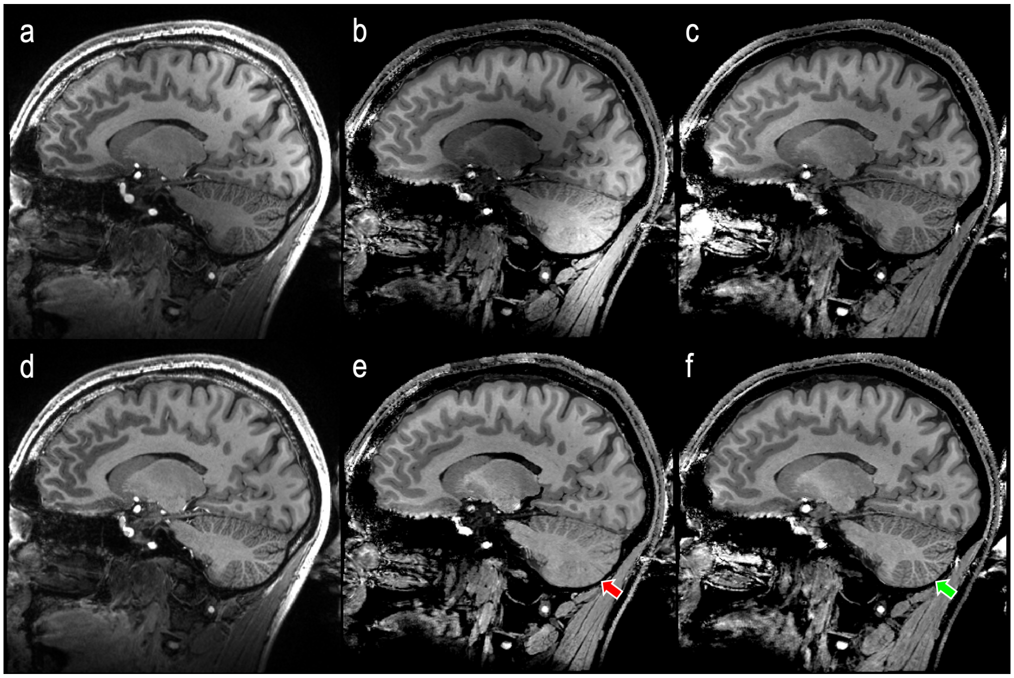

Fig. S2. Sagittal views of MPRAGE (a), MPRAGE*like*, *all* using circular-polarized (non-pTx) excitation (b) and MPRAGE*like*, *all* using UP pTx excitation (c). The bottom row (d-f) shows the same images after N4 correction, demonstrating that N4 correction cannot compensate for the lack of B_{1+} homogeneity in the cerebellum, if excitation was not homogenized (red arrow), as opposed to homogenization through universal pTx pulses (green arrow). The MPRAGE*like*, *all* images shown here were obtained using λ=200 for background noise suppression.

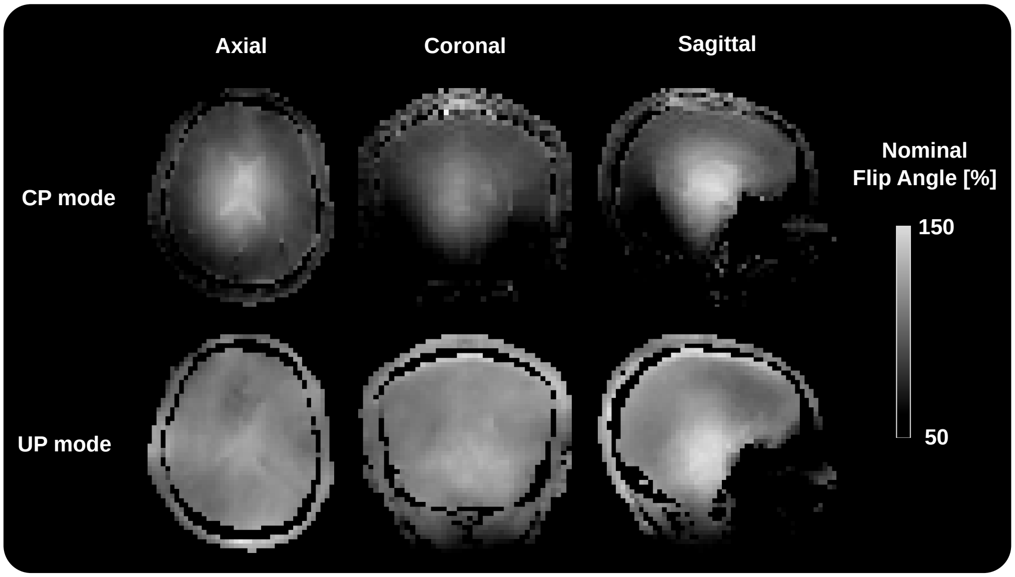

Fig. S3. Visual comparison of the B_{1+} homogeneity between the 1 Tx (circularly-polarized or CP) and pTx (Universal Pulses or UP) modes for one example subject. Universal pulses were used for all sequences in this study resulting in a substantial improvement in transmit homogeneity compared to the CP mode where significant signal dropouts are observed in the cerebellum and temporal lobes. Relative B_{1+} values reported for the UP mode were in vast majority between 80% and 125% whereas values as low as 25% were observed for the CP mode.

REFERENCES

1. Gunther Helms, Henning Dathe, Peter Dechent. Quantitative FLASH MRI at 3T using a rational approximation of the Ernst equation. *Magnetic Resonance in Medicine: An Official Journal of the International Society for Magnetic Resonance in Medicine.* 2008;59(3):667–672.
2. Helms Gunther, Dathe Henning, Kallenberg Kai, Dechent Peter. High-resolution maps of magnetization transfer with inherent correction for RF inhomogeneity and T1 relaxation obtained from 3D FLASH MRI. *Magnetic Resonance in Medicine: An Official Journal of the International Society for Magnetic Resonance in Medicine.* 2008;60(6):1396–1407.
3. Marques José P, Kober Tobias, Krueger Gunnar, Zwaag Wietske, Moortele Pierre-François, Gruetter Rolf. MP2RAGE, a self bias-field corrected sequence for improved segmentation and T1-mapping at high field. *Neuroimage.* 2010;49(2):1271–1281.
